# A 15-Year Retrospective Review of Ciguatera in the Madeira Islands (North-East Atlantic, Portugal)

**DOI:** 10.3390/toxins15110630

**Published:** 2023-10-27

**Authors:** Pedro Reis Costa, Catarina Churro, Susana Margarida Rodrigues, Bárbara Frazão, Miguel Barbosa, Lia Godinho, Lucía Soliño, Viriato Timóteo, Neide Gouveia

**Affiliations:** 1 IPMA—Portuguese Institute of the Sea and Atmosphere, Rua Alfredo Magalhães Ramalho 6, 1495-165 Lisbon, Portugal; catarina.churro@ipma.pt (C.C.); srodrigues@ipma.pt (S.M.R.); barbara.frazao@ipma.pt (B.F.); miguel.barbosa@ipma.pt (M.B.); lia.godinho@ipma.pt (L.G.); luciasolino@gmail.com (L.S.); 2 CCMAR—Centre of Marine Sciences, University of Algarve, Campus of Gambelas, 8005-139 Faro, Portugal; 3 CIIMAR—Interdisciplinary Centre of Marine and Environmental Research, University of Porto, Terminal de Cruzeiros do Porto de Leixões, Av. General Norton de Matos, s/n, 4450-208 Porto, Portugal; 4 Laboratório Regional de Veterinária e Segurança Alimentar, Caminho das Quebradas de Baixo nº 79, S. Martinho, 9000-254 Funchal, Portugal; viriato.timoteo@madeira.gov.pt (V.T.); neide.gouveia@madeira.gov.pt (N.G.)

**Keywords:** ciguatoxins, *Gambierdiscus*, Macaronesia, ciguatera, seafood poisoning

## Abstract

The first ciguatera fish poisoning (CFP) in Portugal dates from 2008 when 11 people reported CFP symptoms after consuming a 30 kg amberjack caught around the Selvagens Islands (Madeira Archipelago). Since then, 49 human poisonings have been reported. The emergence of a new threat challenged scientists and regulators, as methods for toxic microalgae analyses and ciguatoxin (CTX) detection were not implemented. To minimise the risk of ciguatera, the Madeira Archipelago authorities interdicted fisheries in Selvagens Islands and banned the capture of amberjacks weighing more than 10 kg in the entire region of Madeira Archipelago. The accurate identification and quantification of the benthic toxin-producing algae species spreading to new areas require efforts in terms of both microscopy and molecular techniques. Two ciguatera-causing dinoflagellates, *Gambierdiscus excentricus* and *Gambierdiscus australes*, were identified in the Madeira Island and Selvagens sub-archipelago, respectively. Regarding the CTX analysis (N2a cell-based assay and LC-MS) in fish, the results indicate that the Selvagens Islands are a ciguatera risk area and that fish vectoring CTX are not limited to top predator species. Nevertheless, advances and improvements in screening methods for the fast detection of toxicity in seafood along with certified reference material and sensitive and selective targeted analytical methods for the determination of CTX content are still pending. This study aims to revise the occurrence of ciguatera cases in the Madeira Archipelago since its first detection in 2008, to discuss the risk management strategy that was implemented, and to provide a summary of the available data on the bioaccumulation of CTX in marine fish throughout the marine food web, taking into consideration their ecological significance, ecosystem dynamics, and fisheries relevance.

## 1. Introduction

New toxins or toxins that are traditionally known from other regions are emerging in European waters, posing new threats to seafood safety and raising new challenges to authorities and agencies with responsibilities at the official control level. From the list of new toxins and compounds with a potential impact on seafood safety, the occurrence of ciguatoxins (CTXs) is of particular concern.

Ciguatoxins and their precursor metabolites are produced by benthic dinoflagellates of the genera *Gambierdiscus* and *Fukuyoa* [1,2]. Originally considered restricted to endemic ciguatera fish poisoning (CFP) regions within the tropical Indo-Pacific and the Caribbean Seas, the occurrence and spread of these dinoflagellates to sub-tropical and temperate regions is now a reality with trends in climate warming seemingly playing a pivotal role [3,4]. Consequently, the effects of climate change have facilitated the colonisation of new areas or enhanced the performance of existing species, and by means of intensified monitoring efforts, toxic dinoflagellates associated with CFP have been detected in subtropical-temperate regions, such as the Canary Islands, Madeira Archipelago, and the Mediterranean Sea [4,5,6,7,8,9].

The epiphytic nature of *Gambierdiscus*/*Fukuyoa* spp. makes herbivorous fish and grazers particularly prone to accumulate the toxins. Nevertheless, potent CTX derivatives can also be found in omnivorous species and top predator fish due to toxin biotransformation in metabolic processes that are not yet fully understood [1,2,10]. 

Although ciguatoxins are recognised as highly potent neurotoxins, they are still poorly regulated, in contrast to the “classic” toxins that have a consistent EU regulation with standard operating procedures expressing the sampling frequency, reference detection methods, and safety limits [11,12]. According to the Regulation (EC) No 853/2004, the fishery products containing ciguatoxins or muscle-paralyzing toxins, must not be placed on the market. This regulation would be sufficient to protect European consumers from the importation of contaminated fish from endemic regions. Still, it is clearly insufficient to control its recent natural occurrence in European waters. Surprisingly, the first known CFP outbreak in the European Union involving fish caught in European waters occurred the same year as the publication of this EU regulation. The outbreak affected five persons after the consumption of a 26 kg longfin yellowtail *Seriola rivoliana* from the Canary Islands in 2004 [13]. CFP episodes in the Canary Islands (Spain) and then in the Madeira Archipelago (Portugal) [13,14] raised attention concerning the emergence of new marine toxins that were not traditionally monitored by seafood safety authorities. 

## 2. Ciguatera Poisonings in Madeira Archipelago

The first reported outbreak in Portugal dates from 2008 when 11 crew members of a total of 16 persons in a fishing boat reported CFP symptoms after consuming a 30 kg amberjack (*Seriola* sp.) caught around the Selvagens Islands [14,15]. The symptoms reported by the crew members matched with symptoms previously reported by nature wardens of the natural park of the Selvagens Islands back in 2007. The symptoms, mostly neurologic issues, appeared hours or days after the ingestion, which hindered a clear association with the type of food that caused the poisoning, having initially suspected the supplies taken on the ship from Madeira Island, and renewed only every three weeks when the guards rotated off duty. After the acute outbreak in July 2008, it was noticed that the intoxication of the nature wardens was due to the ingestion of locally caught fish and not subsistence provisions. CP was retrospectively diagnosed in the six wardens who had consumed fish caught locally, including amberjack, parrotfish (*Sparisoma cretense*), blacktail comber (*Serranus atricauda*), barred hogfish (*Bodianus scrofa*), grey triggerfish (*Balistes capriscus*), and red porgy (*Pagrus pagrus*) [15]. The duration of the neurological symptoms lasted between 0.5–1.5 months [15]. Also in 2008, CFP symptoms were observed in 20–30 people who consumed lesser amberjack (*Seriola rivoliana*) purchased in the Canary Islands but captured in the Selvagens Islands [16]. 

Considering the higher number of CFP cases associated with the consumption of fish captured in the Selvagens Islands, this location is seen as the most relevant ciguatera-prone area in Portugal. In recent years, the Selvagens nature wardens and other inhabitants adapted their diet, which was initially rich in highly nutritional fresh fish, to salted and canned industrial manufactured products. To date, 49 cases of human intoxication have been reported in Portugal (Madeira Island and Selvagens sub-archipelago). This number may be regarded as a low value, suggesting that CFP is not a common intoxication, but ciguatera is not a mandatory notifiable disease in Madeira Archipelago, which certainly leads to several unreported cases.

## 3. The Particular Case of Selvagens Islands

Considering that most of the CTX-contaminated fish were detected in Selvagens Islands and not in Madeira Island [17,18], it is important to better understand the specificities of this region. Since the CTX-contaminated fish that caused the first CFP in Portugal was captured in the Selvagens Islands [14], and several other CFP cases reported both in Portugal and Spain were associated with fish from these islands [16,17,18], the Selvagens Islands may, in fact, be seen as a hotspot for ciguatera in the European Union: the Selvagens Islands are of volcanic origin and are located between Madeira Island and the Canary Islands, 600 km west from the African coast of Morocco, a highly productive coastal upwelling system [19]. These constitute the oldest marine protected area in Portugal, classified as a nature reserve in 1971 to protect the world’s largest breeding colony of Cory’s shearwater, *Calonectris borealis,* and other threatened seabird species in the NE Atlantic. The protection area was extended to the maritime area around the islands comprising a total of 95 km^2^, and delimited by the 200 m depth bathymetry line. Despite this being a protected marine area, some exceptions to the general ban on fisheries in the Selvagens Islands were initially allowed, such as spearfishing and angling; however, after the 2008 outbreak a total ban on fisheries was issued due to the risk of ciguatera poisoning. 

Recently, the Madeira Government revised the legal regime of the Selvagens Islands Nature Reserve. Because of the limit of the marine protected area associated with the 200 m bathymetry line can be reached within a few hundred meters from the coastline due to the narrow and steep insular shelf, the marine-protected area was extended up to 12 nautical miles, with a total area of 2677 km^2^ that includes the entire land area of the Selvagem Grande and Selvagem Pequena islands, and a series of adjacent islets, making it the largest fully protected marine area in Europe and the most intact ecosystem in the North-East Atlantic [20].

**Figure 1:**
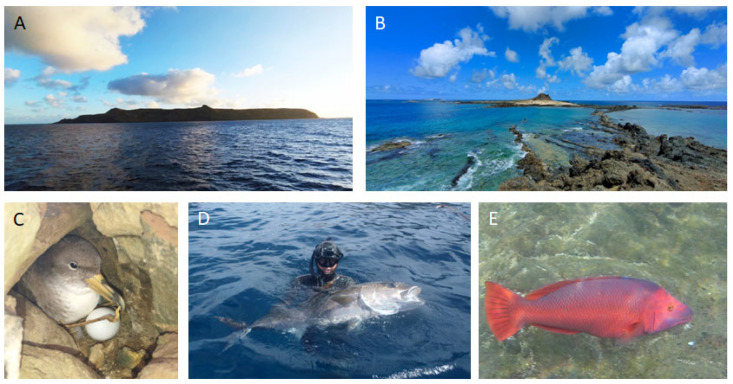
View of the Selvagem Grande (**A**) and Selvagem Pequena (**B**), and iconic wildlife animals, such as the Cory’s shearwater *Calonectris borealis* (**C**), the greater amberjack *Seriola dumerilli* (**D**), and the barred hogfish *Bodianus scrofa* (**E**).

A few studies have pointed out that the waters surrounding the Selvagens Islands are home to some of the most well-preserved oceanic habitats and some of the last remaining intact marine ecosystems in the eastern Atlantic. The Selvagens Islands have been designated by the National Geographic Society as one of most pristine ocean sites in existence [21] (Figure 1). The few coastal underwater visual fish censuses that have been carried out to date have indicated an impressive abundance and diversity [22,23]. Friedlander and colleagues [23] reported a fish biomass 3.2 times larger at Selvagens Islands than at Madeira Island, and a biomass of top predators more than 10 times larger. Several commercially important species and ciguatera risk species are more common and of a larger size in the Selvagens Islands than at Madeira Island. The amberjack *Seriola dumerili* seems to be one of the most important species by weight at Selvagens Islands and *S*. *rivoliana* was found to be seven times more abundant in the Selvagens Islands compared to Madeira Island. Barred hogfish (*Bodianus scrofa*) was 3.4 times more abundant by weight in the Selvagens Islands compared to Madeira Island [23]. In contrast to the heavily populated Madeira or Canary Islands, the Selvagens Islands harbour a well-preserved marine biota where the impacts of human activity and pressures from fisheries pressure are practically non-existent, and where fish may live longer, reach higher sizes, and possibly accumulate higher CTX levels.

## 4. Occurrence and Distribution of *Gambierdiscus*

The marine benthic dinoflagellate genus *Gambierdiscus* was first discovered in the late 1970s in the Gambier Islands, French Polynesia, when it was recognised as the source of ciguatoxins and linked to the recurrent ciguatera food poisoning episodes [24]. The isolate was described as *G. toxicus*, the type species [25]. The *Gambierdiscus* genus remained a monotypic taxon for a considerable period of time, but currently, at least 21 *Gambierdiscus* and 4 *Fukuyoa* species have been described with the aid of both morphological and molecular marker information [26,27,28,29]. In 1956, *Gambierdiscus* was first described in the NE Atlantic as *Goniodoma*, in Cape Verde [30], then proposed by Fraga et al. (2011) [6] as a *Gambierdiscus* species, and re-described as a new species, *G. silvae* [31]. Cape Verde comprises the southern islands of the Macaronesia region, an ecozone that also includes the Canary, Madeira, and Azores Archipelagos. 

**Figure 2:**
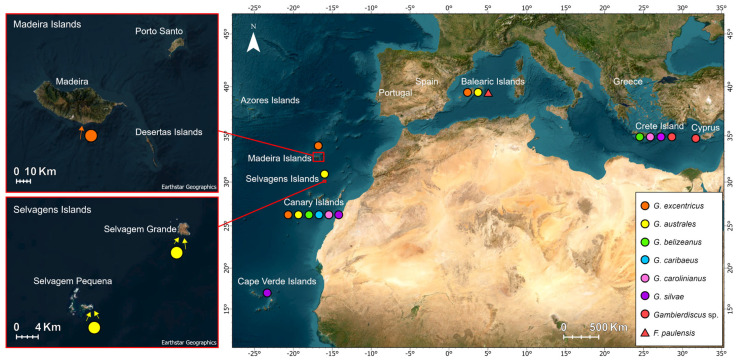
Distribution of *Gambierdiscus* species in the Macaronesia and the Mediterranean Sea, and the location of the Madeira and Selvagens Islands (NE Atlantic) [5,7,8,9,28,35,36,37].

The occurrence and spread of *Gambierdiscus* species and CFP outbreaks have increased in temperate regions [3,32]. Furthermore, ciguatera-causing dinoflagellate *Gambierdiscus* and *Fukyuoa* have been detected in the southern waters of the European Community, such as Canary Islands (Spain) and Madeira Archipelago (Portugal), and in temperate regions of the Mediterranean Sea [6,8,9,31,33,34] (Figure 2).

**Figure 3:**
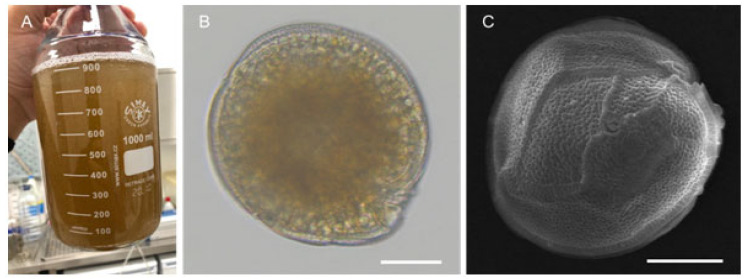
*Gambierdiscus* collected from the Madeira and Selvagens Islands. Culture concentrate (**A**), *Gambierdiscus* sp. isolated from Madeira in light microscopy (scale bar 25 µm) (**B**), and *G. australes* isolated from Selvagens Islands (scale bar 10 µm) (**C**).

Only two species have been reported to date for the Portuguese islands of Madeira and Selvagens sub-archipelago, namely *G. excentricus* and *G. australes*, respectively, contrasting with the diversity of *Gambierdiscus* species observed in the Canary Islands, which includes *G. australes*, *G. caribaeus*, *G. carolinianus*, *G. excentricus*, and *G. silvae* [26,31,38] (Figure 2). *Gambierdiscus australes* was reported in seawater, macroalgae and artificial substrate samples from Selvagens Islands [7,9] and *G. excentricus* from Madeira Island [8] (Figure 3). The first was described from Selvagens Islands by Reverté and colleagues [7] after harvesting them from macroalgae at 0.5–1.5 m deep in the early autumn of 2013. Later in 2018, Godinho and colleagues [9] used mosquito nets, as artificial substrate, to harvest and quantify the *Gambierdiscus* and other toxic benthic dinoflagellates in both Selvagem Grande and Selvagem Pequena. Again, only *G. australes* was retrieved at depths from 3 to 7 m. Cell concentration in the artificial substrate reached 1302 cells at 100 cm^−2^, which is in line with the reports made by Tester and colleagues [39] in Belize and Malaysia, but below the maximum levels reported in the nearby Canary Islands [40]. Both Reverté and colleagues [7] and Tester and colleagues [9] assessed the CTX-like toxicity of *G. australes* strains isolated from the Selvagens Islands by means of Neuro-2a (N2a) cell-based assays (CBAs). The previously determined values of up to 515 fg equiv. CTX1B. cell^−1^ and the former did not exceed 83 fg equiv. CTX1B. cell^−1^. Results from this functional assay may suggest a toxin profile composed of both CTX- and MTX-like compounds. Chemical analysis by liquid chromatography coupled to either low- or high-resolution mass spectrometry (LC-MSMS and HRMS) in six *G. australes* strains from the Balearic Islands (Spain) indicated the presence of maitotoxin-5 (MTX5), 44-methyl gambierone, and gambieric acids C and D, but no CTX precursors were detected [35,41].

On Madeira Island, *G. excentricus* was first described from formaldehyde preserved samples collected in 2008 along the islands’ southern shore [42]. Subsequent opportunistic sampling was carried out in 2014 in Lido, on the south coast of the Madeira Island, which confirmed the occurrence of *G. excentricus* after morphological and phylogenetic analysis [8]. More recently, our research group confirmed the presence of *G. excentricus* on the north coast of Madeira Island, which is heavily exposed to wind-driven wave action and currents, as well as the nearby Desertas Islands and Porto Santo (unpublished data). 

While *Gambierdiscus* strains from the Selvagens Islands were tested for cytotoxicity via the neuroblastoma 2-a (N2a) assay, no data were available for *G. excentricus* from Madeira Island. However, this species exhibited extremely high toxicity in strains isolated from the Canary Islands [43]. Chemical analysis by LC-HRMS showed that maitotoxin-4 (MTX-4) is the main compound produced by *G. excentricus* strains from the Canary Islands, Brazil, and the Caribbean Sea [35,44], leading these authors to suggest that MTX4 may be used as a biomarker for *G. excentricus*. The toxins profile of *Gambierdiscus* from Portuguese waters remains to be characterised by LC–MS/MS or HRMS.

## 5. Ciguatoxicity and Ciguatoxins Profile in Seafood

**Figure 4:**
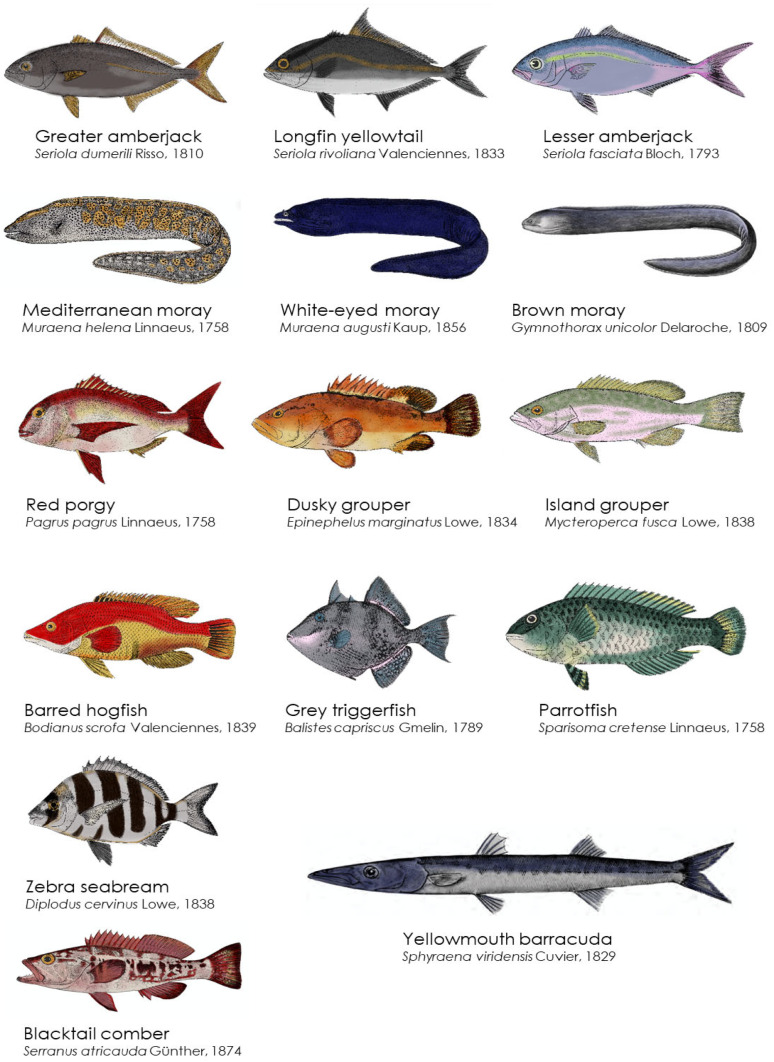
Fish species potentially acting as CTX vectors in Selvagens Islands coastal waters. Fish representations were adapted from [49].

Fish species potentially vectoring CTXs have been captured in Madeira Island and the Selvagens sub-archipelago to assess the risk of CFP (Figure 4). Screening the CTX-like toxicity by N2a cell-based assay (CBA) and toxin determination via sensitive liquid chromatography with tandem mass spectrometry detection (LC–MS/MS), as described in Esteves and colleagues, and Costa and colleagues [45,46], were carried out in recent years. CTXs were mostly found in fish samples from the Selvagens Islands, whilst a few were from Madeira Island (Table 1).

The first poisoning episodes were associated with greater and lesser amberjack (*Seriola dumerilli* and *S. fasciata*), and the analysis indicated extremely high levels of up to 40 µg kg^−1^ by CBA and up to 35 µg kg^−1^ by LC–MS [14]. Differences between the quantitative results of earlier and more recent studies should be associated with advances in analytical chemistry in recent years. The detection of CTX by LC–MS/MS is complex and requires extensive extraction procedures and sensitive instruments. Accurate quantification is rather difficult due to the lack of analytical standards and lack of data on the toxicological potential of many CTX analogues, mainly of the Caribbean type. The reassessment of CTX toxicity and toxins profile characterisation in the fish (*S. fasciata*) caught in 2008 which was implicated in human poisonings [47] revealed a toxicity level of 1.4 µg C-CTX1 eq·kg^−1^, with C-CTX1 the dominant compound, which reached 0.84 µg C-CTX1 kg^−1^ when quantified by LC–MS/MS [48].

Toxicity levels up to 0.76 µg C-CTX1 kg^−1^ were recently assessed in red porgy (*Pagrus pagrus*) from the Selvagens Islands. This fish was analysed after being associated with a CFP episode in the Canary Islands [45,48]. In addition to red porgy, other fish species with intermediate positions in the marine trophic chain, such as zebra seabream (*Diplodus cervinus*) and barred hogfish (*Bodianus scrofa*), showed relevant CTX-like toxicity levels of up to 0.75 µg C-CTX1 kg^−1^ [46]. Screening CTX toxicity by N2a-CBA in a diversity of coastal fish indicated that several species had the potential to act as CTX vectors to humans, which including the parrotfish (*Sparisoma cretense*), the grey triggerfish (*Balistes capriscus*), and the blacktail comber (*Serranus atricauda*), highlighting that ciguatera risk is not exclusively associated with top predators [46]. 

Until recently, the general consensus on ciguatera suggested that top fish predators are the most ciguateric species, either because potent CTX bioaccumulates in the fish metabolism or because of the lipophilic nature of CTX. However, evidence from both traditional knowledge and modern science, including reports from the Selvagens Islands’ nature wardens, suggests that both omnivore and piscivorous fish are toxic to ciguatera [46]. 

In addition to the pelagic environment, an opportunistic sampling of benthic organisms including the starfish *Ophidiaster ophidianus* from the southeast coast of the Madeira Island revealed the presence of mass spectrometry signals and ion fragmentation patterns associated with CTX [50], highlighting the need to further investigate CTX transfer dynamics in bottom-dwelling organisms.

The Caribbean-CTX1 (C-CTX1) was found to be the main compound determined in fish [16,17] which, due to the lack of standards, needed to be further confirmed by high-resolution mass spectrometry (LC-HRMS) [51]. When functional assays, namely the cytotoxicity assay neuroblastoma 2-a (N2a), were used in combination with LC–MS/MS, the presence of C-CTX1 derivatives or other CTX metabolites was suggested [48,52], highlighting the need for deeper analyses and further research to characterise the complete profile of ciguatoxins. As such, several ciguatoxins analogues were identified and quantified in barred hogfish (*Bodianus scrofa*) by capillary LC-HRMS: 17-hydroxy-50,51-didehy-dro-C-CTX3, C-CTX3/4, 50,51-didehydro-C-CTX3, C-CTX1a, 56-methoxy-C-CTX1, and 17-hydroxy-56-methoxy-C-CTX1 [52].

The dataset of CTX analysis carried out in recent years to fish from the Portuguese coast suggests a moderate-to-high risk of CFP according to the international guidelines and recent research studies. The pioneer studies carried out by Lewis and colleagues during the 1990s investigating the in vivo lethal dose of Pacific and Caribbean CTX indicated a much lower potency compared to the later ones [53], and the US Food and Drug Administration established guidance levels for ciguatoxins, with a ten-fold less conservative level for fish contaminated with Caribbean CTX (0.1 µg C-CTX1 eq. kg^−1^) than Pacific CTX (0.01 µg P-CTX1 eq. kg^−1^) [54]. However, recent studies suggest similar potencies between Pacific ciguatoxins and Caribbean congeners, as based on in silico simulations and functional cell studies, such as the N2a-MTT assay and electrophysiological recordings [55]. Further evidence highlighting the need to revise the relative potency of the CTX congeners was raised by a Japanese research group after a series of functional analyses of Pacific CTX congeners [56]. Finally, it is relevant to keep in mind that, most of all, the lack of certified reference standards for the accurate quantification of the toxins in seafood and the poor knowledge of the toxicity potential of each ciguatoxin hampers the evaluation of the risk of ciguatoxins in fish to consumers.

**Table 1:** CTX (μg kg^−1^) levels detected in fish samples from Portuguese waters.

Species	Common Name	Location of Capture	Weight (kg)	Date	Tissue	N2a-CBA	LC-MSMS/HRMS		Reference
							Concentration	Toxins	
*Seriola rivoliana* *	Longfin yellowtail	Selvagens Islands	not specified	2008	Flesh	0.17 µg C-CTX1 eq. kg^−1^	Not quantified	C-CTX1	[16]
*Seriola fasciata* *	Lesser amberjack	Selvagens Islands	not specified	2008	Flesh	up to 6.23 µg C-CTX1 eq. kg^−1^	Not tested	Not tested	[47]
*Seriola* sp. *	Amberjack	Selvagens Islands	not specified	2009	Flesh	0.08 µg C-CTX1 eq. kg^−1^	Not quantified	C-CTX1	[16]
*Seriola fasciata* **	Lesser amberjack	Selvagens Islands	10.0	March 2009	Tail muscle	40.6 µg kg^−1^	35.29 µg kg^−1^	CTX-1B at *m*/*z* 1111.6, CTX-3C at 1023.5 *m*/*z*, CTXs at *m*/*z* 1040.6 and 1141.6	[14]
*Seriola dumerili* **	Greater amberjack	Selvagens Islands	70.0	April 2009	Several tissues (tail, head, ventral, liver)	37.3–45.1 µg kg^−1^	33.29–54.35 µg kg^−1^		[16]
*Seriola fasciata* *	Lesser amberjack	Selvagens Islands	37.0	November 2008	Flesh	1.4 µg C-CTX1 eq. kg^−1^	0.84 µg C-CTX1 eq. kg^−1^	C-CTX1 and three C-CTX congeners of *m*/*z* 1157, *m*/*z* 1127 and *m*/*z* 1123	[45]
*Gymnothorax unicolor*	Brown moray	Selvagens Islands	1.2	December 2013	Flesh	0.039 µg CTX1B eq. kg^−1^		CTX at *m*/*z* 1127.6, dihydro-CTX2 analogue at *m*/*z* 1115.6	[57]
*Muraena augusti*	Morey eel	Desertas Islands	1.7	November 2013	Flesh	0.065 µg CTX1B eq. kg^−1^			[57]
*Muraena helena*	Mediterranean moray	Desertas Islands	1.3	November 2013	Flesh	0.083 ± 0.014 µg CTX1B eq. kg^−1^			[57]
*Pagrus pagrus* *	Red Porgy	Selvagens Islands	4.0	December 2016	Flesh	Not tested	0.76 µg C-CTX1 eq. kg^−1^	C-CTX1 and a hydroxyl metabolite at *m*/*z* 1181.7	[48]
*Epinephelus marginatus*	Dusky grouper	Selvagens Islands	19.5	December 2016	Flesh	Not tested	0.05 µg C-CTX1 eq. kg^−1^	C-CTX1	[17]
*Mycteroperca fusca*	Island grouper	Selvagens Islands	4.5	December 2016	Flesh	Not tested	0.25 µg C-CTX1 eq. kg^−1^		[17]
*Bodianus scrofa*	Barred hogfish	Selvagens Islands	1.7–0.8	November 2017	Flesh	Not tested	0.11–0.06 µg C-CTX1 eq. kg^−1^		[17]
*Balistes capriscus*	Grey triggerfish	Selvagens Islands	2.0	November 2017	Flesh	Not tested	0.03 µg C-CTX1 eq. kg^−1^		[17]
*Sparisoma cretense*	Parrotfish	Selvagens Islands	0.9–0.4	September 2018	Flesh	0.006–0.04 µg CTX1B eq. kg^−1^	<LOQ		[46]
*Diplodus cervinus*	Zebra seabream	Selvagens Islands	2.8	September 2018	Flesh	0.37 µg CTX1B eq. kg^−1^	<LOQ	C-CTXs congeners, C-CTX-1157	[46]
*Bodianus scrofa*	Barred hogfish	Selvagens Islands	3.0–1.1	September 2018	Flesh	0.04–0.75 µg CTX1B eq. kg^−1^	0.08–0.48 µg C-CTX1 eq. kg^−1^	C-CTX1	[46]
*Balistes capriscus*	Grey triggerfish	Selvagens Islands	2.6–0.6	September 2018	Flesh	<LOQ–0.06 µg CTX1B eq. kg^−1^	<LOQ–0.09 µg C-CTX1 eq. kg^−1^	C-CTX1	[46]
*Serranus atricauda*	Blacktail comber	Selvagens Islands	0.3–0.2	September 2018	Flesh	0.006–0.02 µg CTX1B eq. kg^−1^	<LOQ		[46]
*Sphyraena viridensis*	Yellowmouth barracuda	Selvagens Islands	6.0–2.2	September 2018	Flesh	up to 0.22 µg CTX1B eq. kg^−1^	up to 0.14 µg C-CTX1 eq. kg^−1^	C-CTX1	[46]

* associated with CFP outbreaks in Spain. ** associated with CFP outbreaks in Portugal.

## 6. Ciguatera Risk Management

Although fish caught in Madeira Island tested negative for CTXs, more extensive sampling is needed to confirm these results, especially considering the recent identification of *Gambierdiscus excentricus* in the island´s coastal waters [8]. The lower incidence of CFP in the Madeira Archipelago concerning the Selvagens sub-archipelago may be related to the higher fishing pressure. Some authors suggested that fishing for small fish may ‘remove’ CTX from the environment, reducing their transference and bioaccumulation to top predators [58]. To minimise the risk of CFP, and without an international official method for CTX analysis nor certified standards, the Madeira Government forbade the commerce of amberjacks and groupers above 10 kg in the Madeira Archipelago. Regarding the Selvagens Islands, which seems to be the most problematic area for ciguatera, extending the marine protected area to 12 nautical miles, where there is a total ban on fishing activities, would highly reduce the chances of introducing contaminated fish and fish products in the market. With these measures, the Madeira Government aims to protect seafood consumers and to comply with the EU directives that state that fishery products containing ciguatoxins must not be placed on the market [11]. However, the regulations exclusively based on fish weight may be ineffective and counterproductive for the fishing sector [18]. It may also induce illegal capture, commerce, and ultimately the consumption of fish that potentiate unreported and untraceable cases of ciguatera poisoning.

In contrast, in the nearby Macaronesian archipelago of the Canary Islands, the government implemented an official control for ciguatoxins in certain fish species, such as amberjacks (*Seriola* spp. ≥ 12 kg), dusky grouper (*Epinephelus marginatus*, ≥12 kg), island grouper (*Mycteroperca fusca*, ≥7 kg), bluefish (*Pomatomus saltatrix*, ≥9 kg), and the wahoo (*Acanthocybium solandri*, ≥35 kg). These fish species, with these weight or above, cannot be placed on the market without a previous negative result by the N2a cell-based assay [59,60]. Finally, ciguatera is a mandatory notifiable disease in the Canary Islands, which allows us to better trace back the contamination of the marine resources or to identify any deviation to the official control contributing to its improvement.

## 7. Perspectives

HABs are natural phenomena occurring for millions of years causing devastating effects in marine animals over geological timescales [61,62], and ciguatera has been known since the times of the earlier Portuguese and Spanish explorers in the Age of Discovery [63]. Although known for centuries, HABs forecasting remains a challenge and very limited studies have contributed to the prediction of the contamination of marine resources [64,65]. The next frontier in the context of HABs and seafood safety is to incorporate newly available technology and scientific knowledge from multiple disciplines.

Selvagens Islands, which are the southernmost point of Portugal, are here highlighted as a key location for carrying out studies on *Gambierdiscus* dynamics, the toxin transfer in the coastal food web, and fish toxin metabolism. Studies involving intensive sampling in these remote islands are needed; however, the best strategy for sampling benthic dinoflagellates is still under debate. The use of an artificial substrate has been seen as the best approach for sampling and quantifying benthic dinoflagellates, but it may be technically complex, requiring divers or scuba-divers, and it is time-consuming as it has to be submerged for 24 h [39,40,66]. The identification of the toxic dinoflagellates at the species level also requires complex microscopic analysis, and in the case of *Gambierdiscus,* should be combined with molecular analysis. Furthermore, the cultivation of *Gambierdiscus* and *Fukuyoa* dinoflagellates is slow due to their low growth rates in artificial culture conditions, without favouring genetic and toxin analysis when requiring a certain amount of biomass. The accurate identification and quantification of toxin-production algae species spreading to new areas, their life stages, and toxicity require efforts in terms of molecular techniques and genetics, whilst cell culturing and routine analysis should be implemented to better characterise the risk of CFP (Figure 1). Azores are the only Macaronesian archipelago where *Gambierdiscus* and or *Fukuyoa* have not yet been detected. Also, the Portuguese and Spanish peninsular regions are considered free of these species but these have already been reported in the Mediterranean Sea and the Balearic Islands [5,35]. Moreover, molecular signals of *Gambierdiscus* genetic material were detected throughout the NE Atlantic from the Iberian Peninsula to the Azores by the Tara Oceans expedition [67], indicating that *Gambierdiscus* can be far more spread in the North Atlantic than previously thought. In fact, LC–MS/MS signals associated with CTX were detected in two starfish species (*Ophidiaster ophidianus*, *Marthasterias glacialis*) from the Azores [50], raising suspicions about the spread of toxic dinoflagellates *Gambierdiscus*/*Fukuyoa* into these waters.

Finally, advances are needed in screening methods for the fast detection of toxicity in seafood as well as sensitive and selective targeted analytical methods for the accurate determination of Caribbean ciguatoxin contents [68]. The development of biosensors for the detection of toxic *Gambierdiscus* and/or *Fukuyoa* dinoflagellate species and toxic fish specimens is of high value for remote islands, such as the Selvagens Islands. A review of the state of the art of biosensors to assess the ciguatera risk has been recently published by Gaiani and colleagues [69]. Of critical importance is the production of the reference material and the availability of standards for Caribbean ciguatoxins [70], essential for the method validation and implementation of the official control. Simultaneously, improved data are needed on the toxicological potency of the different CTXs. Once these tasks are achieved and monitoring programs are implemented in the Madeira Archipelago and other parts of the world, it will be possible to better understand the risk of CFP, the distribution of the ciguatoxins in the environment, and their metabolism by fish species. A comprehensive and multidisciplinary approach fostering collaboration among research institutions, monitoring agencies, fisheries, authorities, healthcare professionals, decision-makers, and the government, is absolutely essential for effective fishery management. Such synergy is pivotal in ensuring accurate risk assessment and safeguarding food security in Portugal.

## Data Availability

Not Applicable.
